# Supplementary material for: Genetic-optimised aperiodic code for distributed optical fibre sensors
Source: Nat Commun. 2020 Nov 13;11:5774. doi: 10.1038/s41467-020-19201-1 (PMC7666181; doi:10.1038/s41467-020-19201-1)
Supplement: Supplementary file 1 — Supplementary Information [file 41467_2020_19201_MOESM1_ESM.pdf]

Supplementary Information

Genetic-optimised aperiodic code for distributed optical fibre sensors

Xizi Sun[1,4], Zhisheng Yang[2,4*], Xiaobin Hong[1*], Simon Zaslawski[2], Sheng Wang[1], Marcelo A. Soto[3], Xia Gao[1], Jian Wu[1] and Luc Thévenaz[2]

[1]State Key Laboratory of Information Photonics & Optical Communications, Beijing University of Posts and Telecommunications, Beijing, 100876, China

[2]EPFL Swiss Federal Institute of Technology, Institute of Electrical Engineering, SCI STI LT, Station 11, CH-1015 Lausanne, Switzerland

[3]Department of Electronics Engineering, Universidad Técnica Federico Santa María, Valparaíso, Chile

[4]These two authors contribute equally: Xizi Sun, Zhisheng Yang

Corresponding author: zhisheng.yang@epfl.ch; xbhong@bupt.edu.cn

Supplementary note 1: Fundamentals of the proposed coding technique

Standard single-pulse systems

Distributed optical fibre sensors implemented in the time domain are all based on the concept of optical time-domain reflectometry (OTDR), in which the sensing fibre is interrogated by an optical pulse launched at the fibre near-end. While the pulse propagates through the fibre, the light backscattered from the pulse is recorded as a function of time and then digitally processed to spatially resolve the local information (e.g. temperature, strain, etc.). Such measured discrete-time signal, shown as purple curve in Supplementary Fig. 1 and designated here as $r_s^m(n)$, is essentially a sampled single-pulse response $r_s(n)$ merged with zero-mean additive noise $e_s(n)$:

$$r_s^m(n) = r_s(n) + e_s(n) \qquad 1 \le n \le N_s \tag{1}$$

where n is an integer variable linearly related to the time t through the sampling rate f_s, as $t = n/f_s$, and N_s is the total number of sampled points representing the data length of $r_s^m(n)$, $r_s(n)$ and $e_s(n)$.

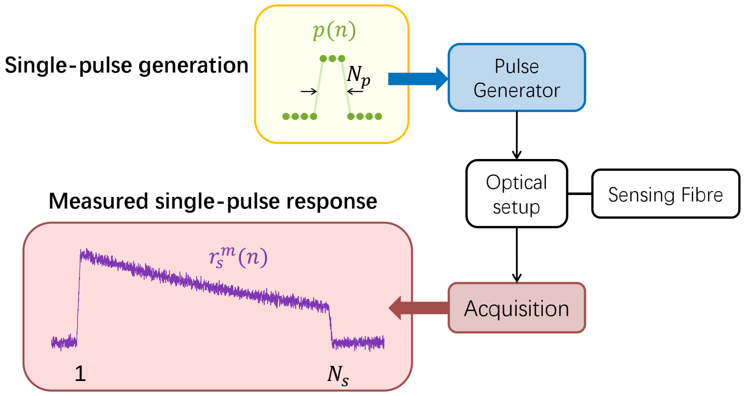

Supplementary Fig. 1. Schematic illustration for the working principle of the single-pulse system.

For a linear time-invariant (LTI) system, the single-pulse response $r_s(n)$ can further be expressed by the *linear convolution*[1] between a single-pulse signal $p(n)$ (see green dots in Supplementary Fig. 1) and the fibre impulse response $h(n)$, so that Supplementary Eq. (1) can be rewritten as:

$$r_s^m(n) = p(n) \otimes h(n) + e_s(n) \tag{2}$$

where sign $\otimes$ denotes linear convolution. Defining a pulse duration T_p and a fibre length L_h, the number of

points in $p(n)$ and $h(n)$ are $N_p = T_p f_s$ and $N_h = 2L_h f_s / v_g$, respectively, where v_g is the pulse group velocity in the fibre. This further yields the relation $N_s = N_p + N_h - 1$ according to the definition of the linear convolution. Supplementary Eq. (2) is often equivalently represented in the discrete-frequency domain as:

$$R_s^M(k) = P(k)H(k) + E_S(k) \qquad -\frac{N_s}{2} \leq k \leq \frac{N_s}{2} - 1 \tag{3}$$

where $R_s^M(k)$, $P(k)$, $H(k)$ and $E_S(k)$ are the respective N_s-point discrete Fourier Transform (DFT)[1] of $r_s^m(n)$, $p(n)$, $h(n)$ and $e_s(n)$, over the *fundamental frequency interval* $[-f_s/2, f_s/2]$ with spacing of f_s/N_s. Note that this process requires to pad $p(n)$ and $h(n)$ with $N_s - N_p$ and $N_s - N_h$ zeros, in order to increase their length to N_s before performing the DFT.

Since $e_s(n)$ represents a single realization of the additive white Gaussian noise (AWGN), which is a *wide-sense stationary* real-valued random process[1], it can be characterized by its variance σ_e^2 expressed as:

$$\sigma_e^2 = \gamma_{ee}(0) = \frac{1}{N_s}\sum_{n=1}^{N_s} e_s(n)^2 = \frac{1}{N_s^2}\sum_{k=-\frac{N_s}{2}}^{\frac{N_s}{2}-1} |E_S(k)|^2 \tag{4}$$

where $\gamma_{ee}(0)$ represents the autocorrelation coefficient of $e_s(n)$ at zero delay.

Proposed pulse-coded system

Similar to the aforementioned single-pulse interrogation, for a given N_c-point coded optical pulse sequence $c_f(n)$ (see Fig. 1 in Results) launched into the sensing fibre, the measured response $r_c^m(n)$ can be respectively described in discrete-time and discrete-frequency domains as:

$$r_c^m(n) = c_f(n) \otimes h(n) + e_c(n) \quad \xleftrightarrow{F} \quad R_c^M(k) = C_F(k)H(k) + E_C(k) \tag{5}$$

where $r_c^m(n)$ and $e_c(n)$ are both N_r-point discrete-time signals ($N_r = N_c + N_h - 1$), and F denotes N_r-point DFT operation. Note that $e_c(n)$ is another single realization of the same AWGN shown in Supplementary Eqs. (1), (2) and (4), so that it shares the same variance σ_e^2 as that of $e_s(n)$. It must be mentioned that this is true only when the thermal noise is dominant, so that the variance of the detection noise is not affected by the power of signal reaching the photo-detector, i.e. not affected by single pulse or code sequence used for the interrogation. This condition can be realised by carefully designing the energy enhancement factor defined in Results, as will be elaborated in Supplementary notes 3 and 4. Knowing that $c_f(n)$ can further be expressed by the linear convolution between $d_f(n)$ (See Fig.1 in Results) and the single pulse $p(n)$, supplementary Eq. (5) can be rewritten as:

$$r_c^m(n) = d_f(n) \otimes p(n) \otimes h(n) + e_c(n) \quad \xleftrightarrow{F} \quad R_c^M(k) = D_F(k)P(k)H(k) + E_C(k) \tag{6}$$

where $d_f(n) = f(n)d(n)$, which can be obtained by retrieving the energy of each optical pulse in $c_f(n)$ measured at the fibre input. This way the following decoding process can be performed:

$$r_s^d(n) = IDFT\left[\frac{R_c^M(k)}{D_F(k)}\right] = IDFT\left[P(k)H(k) + \frac{E_C(k)}{D_F(k)}\right] = r_s(n) + IDFT\left[\frac{E_C(k)}{D_F(k)}\right] \tag{7}$$

resulting in a decoded single-pulse response $r_s^d(n)$ that consists of the targeted single-pulse response $r_s(n)$ and a noise term that is affected by $D(k)$. The measure of whether the decoder $D(k)$ attenuates or magnifies the original input noise is given by the variance of the noise after decoding, which can be characterized based on Supplementary Eq. (4):

$$\sigma_{de}^2 = \frac{1}{N_r^2} \sum_{k=-\frac{N_r}{2}}^{\frac{N_r}{2}-1} \left| \frac{E_C(k)}{D_F(k)} \right|^2 = \sigma_e^2 \frac{1}{N_r} \sum_{k=-\frac{N_r}{2}}^{\frac{N_r}{2}-1} \left| \frac{1}{D_F(k)} \right|^2 = \sigma_e^2 Q \tag{8}$$

where

$$Q = \frac{1}{N_r} \sum_{k=-\frac{N_r}{2}}^{\frac{N_r}{2}-1} \left| \frac{1}{D_F(k)} \right|^2 \tag{9}$$

is defined here as the noise scaling factor that should be minimized to achieve a high SNR. Knowing that $d_f(n)$ is N_x-point upsampling of N_u-point signal $u_f(n)$ as shown in Fig. 1 and expressed here:

$$d_f(n) = \sum_{m=1}^{N_u} u_f(m)\delta(n - mN_x) \qquad 1 \le n \le N_d \tag{10}$$

$|D_F(k)|$ is therefore equivalent to N_x replicas of $|U_F(k)|$, as shown in Supplementary Fig. 2 and proved here:

$$D_F(k) = \sum_{n=1}^{N_r} d_f(n) e^{-i2\pi n \frac{k}{N_r}} = \sum_{n=1}^{N_r} \sum_{m=1}^{N_u} u_f(m)\delta(n - mN_x)\, e^{-i2\pi n \frac{k}{N_r}} = \sum_{m=1}^{N_u} u_f(m) e^{-i2\pi m N_x \frac{k}{N_r}} \tag{11}$$

It is clear that the mean value of $|1/D_F(k)|^2$ is identical to that of $|1/U_F(k)|^2$ regardless of the upsampling points N_x.

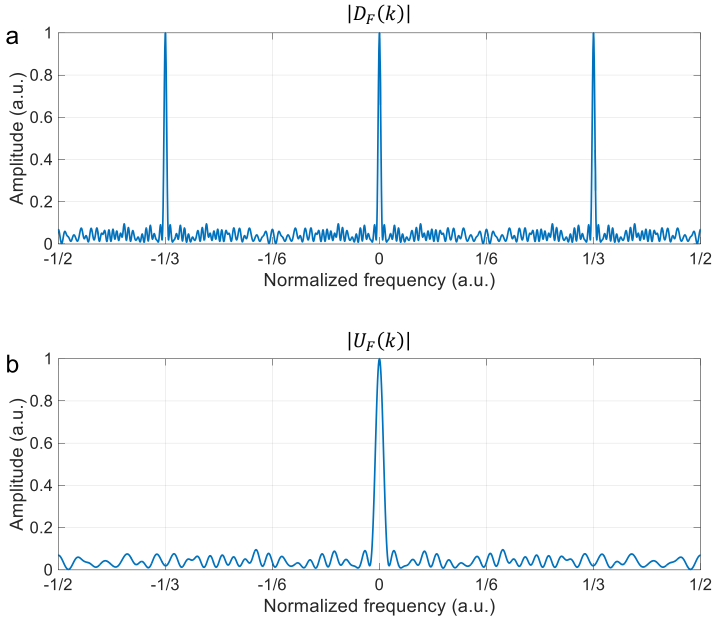

Supplementary Fig. 2. Spectral behaviour of **a** $|D_F(k)|$ and **b** $|U_F(k)|$ for $N_x = 3$.

Through this relation, the noise scaling factor Q can be rewritten as:

$$Q = \frac{1}{N_r} \sum_{k=-\frac{N_r}{2}}^{\frac{N_r}{2}-1} \left| \frac{1}{U_F(k)} \right|^2 \tag{12}$$

Given the energy enhancement factor $F_E = \sum_{n=1}^{N_u} u_f(n)$ and the code length N_u, in $|U_F(k)|^2$ there is a fixed main lobe centred at $k = 0$ with a peak value of $|U_F(0)|^2 \equiv F_E^2$, covering the frequency interval $[-N_r/(2N_u), N_r/(2N_u)]$, as shown in Supplementary Fig. 3(a). This property results in a fixed dip at the centre

of $|1/U_F(k)|^2$, with an approximate power (integrated value over the mentioned frequency interval) of $3N_r/(2F_E^2 N_u)$, as shown by the shaded area in Supplementary Fig. 3(b).

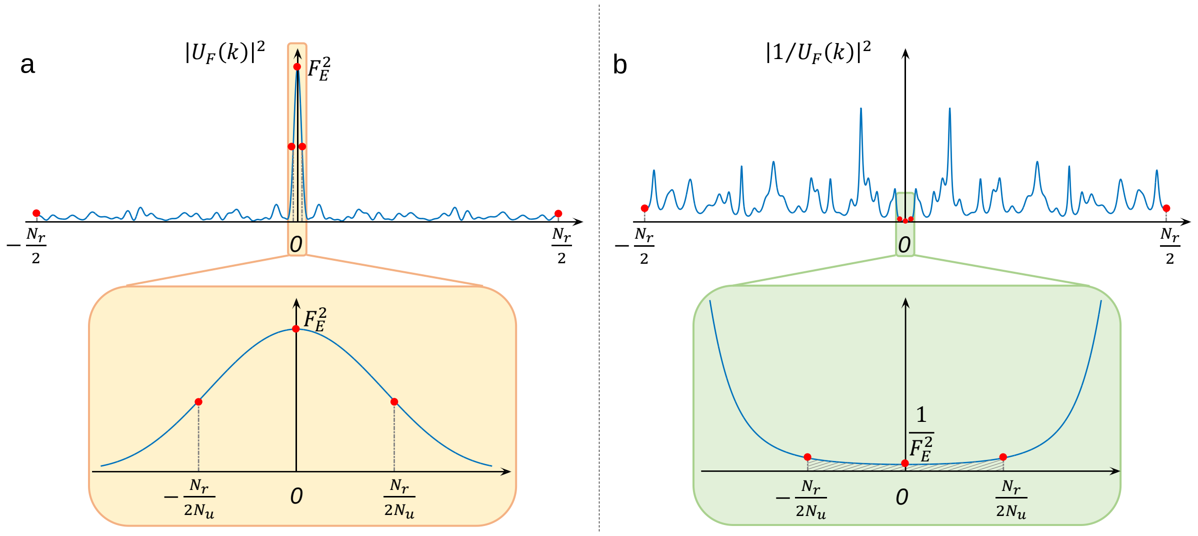

Supplementary Fig. 3. Spectral behaviour of **a** $|U_F(k)|^2$ and **b** $|1/U_F(k)|^2$.

Since Q equals to the mean value of $|1/U_F(k)|^2$ (blue curve in Supplementary Fig. 3(b)), it can be decomposed as:

$$Q \approx \frac{3}{2F_E^2 N_u} + \frac{2}{N_r} \sum_{k=\frac{N_r}{2N_u}}^{\frac{N_r}{2}-1} \left| \frac{1}{U_F(k)} \right|^2 \tag{13}$$

where the first right-hand term represents the mean value of the shaded area in Supplementary Fig. 3(b), which remains fixed for given F_E and N_u (i.e. independent of the detailed code distribution); the second right-hand term represents the mean value outside the shaded area, which depends on the detailed code distribution and is more critical for the determination of the minimum value of Q. Applying *Cauchy inequality* to the second right-hand term of Supplementary Eq. (13):

$$\frac{4}{N_r^2} \sum_{k=\frac{N_r}{2N_u}}^{\frac{N_r}{2}-1} \left| \frac{1}{U_F(k)} \right|^2 \sum_{k=\frac{N_r}{2N_u}}^{\frac{N_r}{2}-1} |U_F(k)|^2 \geq \frac{4}{N_r^2} \left| \sum_{k=\frac{N_r}{2N_u}}^{\frac{N_r}{2}-1} \frac{1}{U_F(k)} U_F(k) \right|^2 = \left(1 - \frac{1}{N_u}\right)^2 \tag{14}$$

and making use of the equality given by the *Parseval's theorem*[1]:

$$\frac{2}{N_r} \sum_{k=\frac{N_r}{2N_u}}^{\frac{N_r}{2}-1} |U_F(k)|^2 = F_E - \frac{F_E^2}{N_u} \tag{15}$$

the following relation can be retrieved:

$$\frac{2}{N_r} \sum_{k=\frac{N_r}{2N_u}}^{\frac{N_r}{2}-1} \left| \frac{1}{U_F(k)} \right|^2 \geq \frac{(N_u - 1)^2}{F_E N_u^2 - F_E^2 N_u} \approx \frac{N_u - 2}{F_E N_u - F_E^2} \tag{16}$$

which further results in:

$$Q \geq \frac{3}{2F_E^2 N_u} + \frac{N_u - 2}{F_E N_u - F_E^2} \tag{17}$$

Defining $N_u = mF_E$, Supplementary Eq. (17) can be rewritten as:

$$Q \geq \frac{3}{2mF_E^3} + \frac{mF_E}{mF_E^2 - F_E^2} - \frac{2}{mF_E^2 - F_E^2} \approx \frac{m}{(m-1)F_E} \qquad for \quad m > 1 \ and \ F_E > 10 \tag{18}$$

This approximation requires $F_E > 20$, which is a reasonable condition to make the implementation of coding techniques meaningful for distributed sensors (sizeable SNR improvement). Note that this minimum value of Q can be reached only if the off-peak spectral region of $|U_F(k)|^2$ (see supplementary Fig. 3) is perfectly flat as required by the *Cauchy inequality* in Supplementary Eq. (14), which is theoretically impossible to obtain[2]. However, the flatter the spectrum, the closer between Q and the right-hand term of Supplementary Eq. (18), for any given F_E and m. Taking this into account and based on the expression of the coding gain (i.e. $G_c = \sqrt{1/Q}$, as defined in Results), the following relation can be obtained:

$$G_c < \sqrt{\frac{F_E(m-1)}{m}} \tag{19}$$

in which the right-hand term represents the theoretical maximum of the coding gain G_c.

Supplementary note 2: Distributed genetic algorithm (DGA)

In order to evaluate the efficiency and effectiveness of the proposed DGA method, a simple brute-force method has been implemented for comparison, targeting a unipolar binary code sequence with total bit number $N_u = 120$ and energy enhancement factor $F_E = 40$. More than 100 million sequences are randomly generated in a 10-hour brute-force searching, and the corresponding probability distribution of coding gain $G_c = \sqrt{1/Q}$ is shown in Supplementary Fig. 4, where the red vertical line represents the standard reference coding gain $G_r = \sqrt{F_E/2} \approx 4.47$. Results show that most values of G_c are less than 3 and clustered around 1.9, while the largest G_c reaches only 3.84 that is still much smaller than G_r, indicating that the brute-force method is impractical to search for an acceptable sequence that can approach the coding gain provided by conventional codes (e.g. Simplex and Golay).

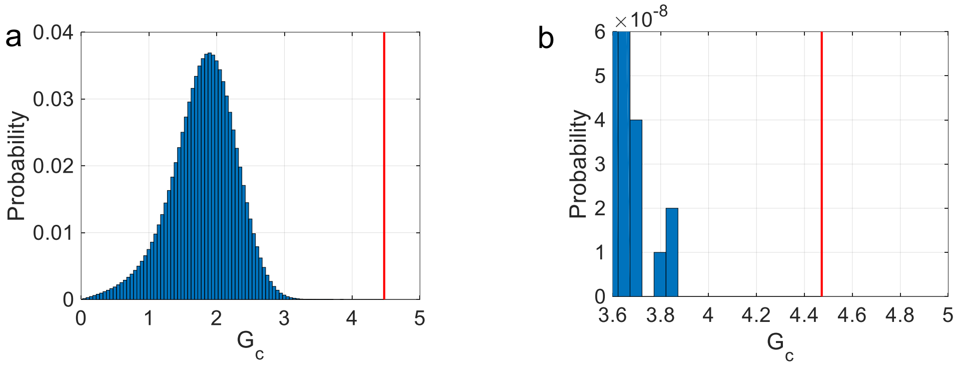

Supplementary Fig. 4. a probability distribution of G_c resulted from 10-hours brute-force searching and **b** probability of G_c distributed from 3.6 to 5 (zoom-in of Supplementary Fig. 4a).

Compared with the brute-force method, DGA has been demonstrated as an effective way to search the optimal/sub-optimal sequence. Supplementary Fig. 5 shows the G_c distribution searched by DGA at the initial generation and 1st, 3rd, 16th, 74th, and 144th iterations, in which each figure contains an inset showing a zoom-in ranging from 2.972 to G_r. It can be found that the G_c distribution in the initial generation is similar to the one shown in Supplementary Fig. 4 due to the same random initialization process. This distribution changes in the evolutionary process, and eventually converges at an optimal $G_c = 4.35$ that is 97% of G_r (0.12 dB less than G_r). Whilst obtaining much better performing G_c, the computational time of DGA searching is ~135 minutes, being order-of-magnitude more time-efficient than the brute-force method.

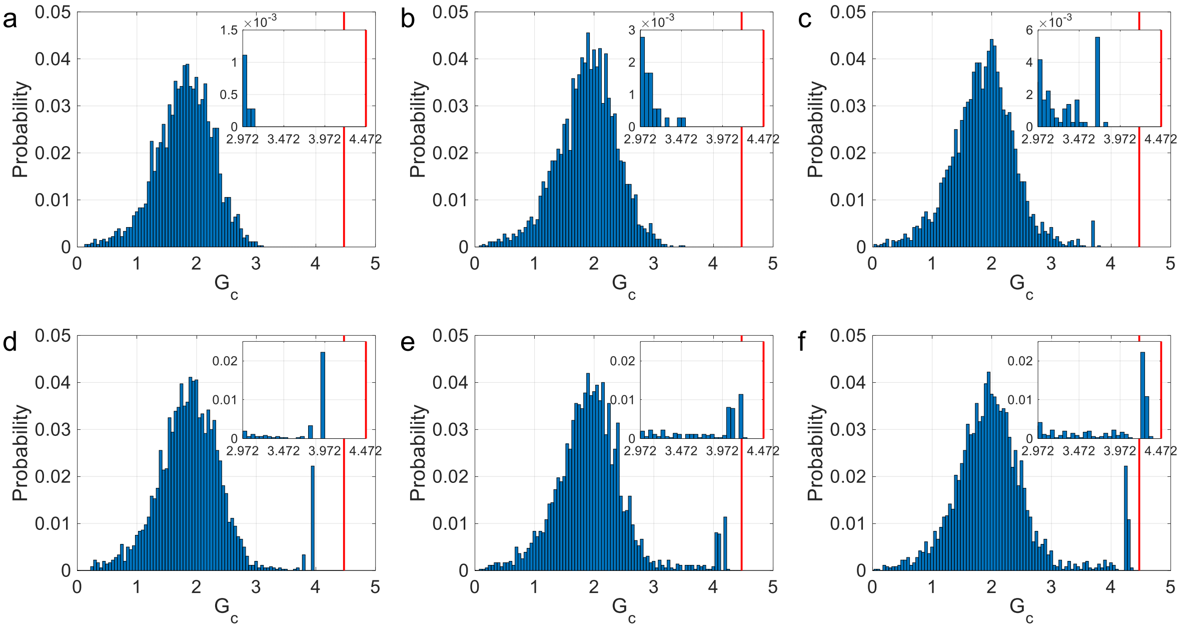

Supplementary Fig. 5. DGA-enabled G_c distribution of **a** initial generation and **b** 1st, **c** 3rd, **d** 16th, **e** 74th, **f** 144th iterations. All insets show a zoom-in of the G_c distributed from 2.972 to G_r.

Supplementary note 3: Noise analysis for unipolar coded BOTDA

In this note the impact of detection noise on general unipolar coded-BOTDA systems is analytically modelled, aiming to provide a quantitative guideline for the optimisation of the energy enhancement factor F_E (defined in Results) under given experimental conditions. It turns out that some Brillouin-gain-dependent noises that are negligible in long-range single-pulse BOTDA, may however be significantly enhanced (even becoming dominant) in unipolar coded-BOTDA due to the large cumulated Brillouin gain. This noise enhancement may substantially compromise the SNR improvement brought by the theoretical coding gain G_c, depending on the value of F_E. In other words, F_E must be properly designed to secure the system operating at a desired G_c.

The analysis is here performed based on the setup shown in Fig. 5, in which a polarization scrambler is employed to mitigate the impact of polarization pulling effects[3,4] and a pre-amplifier (EDFA2) is used to enhance the SNR[5]. The noise model is established by assuming that the BFS profile is uniform, corresponding to the worst-case scenario, i.e., the negative impact of the Brillouin-gain dependent noises is the most detrimental. Considering both the optical noise and detector noise, for the ξth single acquisition (i.e. non-averaged), the photocurrent of the detected optical signal (raw coded BOTDA trace) at the Brillouin resonance can be expressed as[3]:

$$I(t,\xi) = \eta A P_s^{DC} \exp[-G(t,\xi)] + e_{PD}(t,\xi) + e_{s-\mathrm{ASE}}(t,\xi) + e_{s-SpBS}(t,\xi) \tag{20}$$

where t stands for the sampling time, $\eta \approx 1$ A/W is the responsivity of the photodiode, A is the EDFA gain, P_s^{DC} is the power of DC-probe entering the EDFA; e_{PD}, $e_{s-\mathrm{ASE}}$ and e_{s-SpBS} are photo-detection current noise, signal-ASE beating noise and signal-SpBS beating noise, respectively; $G(t,\xi)$ represents the net Brillouin gain at the Brillouin resonance provided by all pulses in the coding sequence, expressed as:

$$G(t,\xi) = \sum_{i=1}^{N_u} g_B(t)\, u_f(i) cos^2\varphi(i,t,\xi) \tag{21}$$

where $g_B(t)$ is the single-pulse Brillouin gain at the Brillouin resonance, N_u and $u_f(i)$ denote the code bit number and the amplitude of the ith coded pulse, respectively, $\varphi(i,t,n)$ is the relative polarization angle between the ith coded pulse and the probe wave, which is randomly distributed over the interval

$[0, \pi]$. To retrieve such net Brillouin gain to reconstruct the BGS, the logarithmic normalization[3] is applied on Supplementary Eq. (20), resulting in:

$$G_{retrieved}(t,\xi) = \ln\left[\frac{I(t,\xi)}{\eta A P_s^{DC}}\right] = \ln\left\{\exp[-G(t,\xi)] + \frac{e_{PD}(t,\xi) + e_{s-\mathrm{ASE}}(t,\xi) + e_{s-SpBS}(t,\xi)}{AP_s^{DC}}\right\}$$
$$\approx -G(t,\xi) + \frac{e_{PD}(t,\xi)}{AP_s^{DC}\exp[G(t,\xi)]} + \frac{e_{s-\mathrm{ASE}}(t,\xi)}{AP_s^{DC}\exp[G(t,\xi)]} + \frac{e_{s-SpBS}(t,\xi)}{AP_s^{DC}\exp[G(t,\xi)]} \tag{22}$$

whose repeatability is affected by 4 noise sources present in the 4 terms, respectively:

I) Polarization noise (presented in the first term of Supplementary Eq. (22)), which results from the polarization fading effect as described by Supplementary Eq. (21) and can be characterized by its standard deviation (STD) as:

$$\sigma_{pol}(t) = K\sqrt{\overline{\mathrm{G}(t,\xi)^2} - \overline{\mathrm{G}(t,\xi)}^2} \approx \frac{KG(t)}{\sqrt{2F_E}} \tag{23}$$

where $G(t)$ is the mean accumulated linear Brillouin gain at the Brillouin resonance, which does not suffer from polarization noise. Mathematically, $G(t) = \overline{G(t,\xi)} = F_E g_B(t)/2$, where F_E is the energy enhancement factor. Note that K is a scaling factor that accounts for the longitudinal fluctuations of the local Brillouin gain standard deviation caused by rotations of the local polarization principle axis. Such phenomenon can be visualized in single-pulse BOTDA, as exemplified in Supplementary Fig. 6(a), where the Brillouin gain at some fibre positions are always close to $g_\mathrm{B}/2$, regardless of the pump pulse polarization state. This variability can be quantified by the variance of the local polarization fading, as shown in Supplementary Fig. 6(b). Different from the single-pulse BOTDA, such a phenomenon is averaged in a coded BOTDA system since the cumulated Brillouin gain $G(t,\xi)$ is attributed to all code pulses that interact with the probe wave at different fibre locations. Here the scaling factor K is obtained by calculating the square root of the ratio between the average variance and maximum variance of the trace shown in Supplementary Fig. 6(b), leading to $K = 0.67$. As demonstrated below, this characterization of K leads to good theoretical noise estimations, matching well with experimental data.

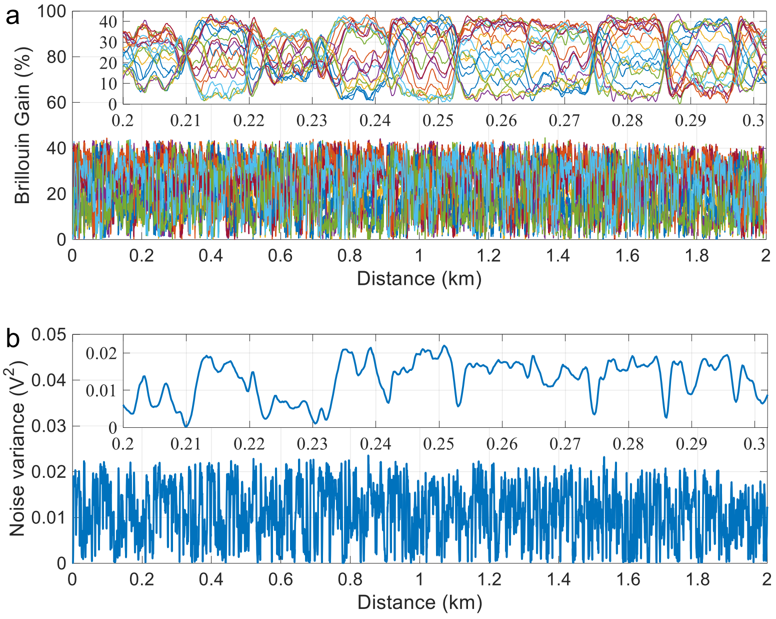

Supplementary Fig. 6. a 20 consecutive normalized single-pulse Brillouin traces obtained without averaging over a 2 km-long SMF at the peak resonance frequency, using a peak pump power around 35 dBm to secure the polarization noise domination. **b** variance of the corresponding local polarization noise. Both insets represent a zoom-in of the respective figure in the range from 0.2 km to 0.3 km.

II) Photo-detection noise (second term in Supplementary Eq. (22)), attributed to thermal noise and shot noise. However, due to the low optical probe power reaching the receiver, the shot noise contribution can usually be ignored. Thus, the noise STD is only related to the thermal noise and can be expressed as:

$$\sigma_{PD}(t) \approx \frac{\sqrt{\sigma_{th}^2}}{P_S^{DC} \exp[G(t)]} \tag{24}$$

where σ_{th}^2 is the thermal noise variance, which can be readily characterized by measuring the photodetector output without input light[5].

III) Signal-ASE beating noise (third term in Supplementary Eq. (22)). The probe signal reaching the receiver beats with the ASE noise introduced by the pre-amplifier, resulting in a noise standard deviation calculated as:

$$\sigma_{s-ASE} \approx \frac{\sqrt{2F_N q(A-1)AP_S^{DC}\exp[-G(t)]\,B_e}}{AP_S^{DC}\exp[-G(t)]} \approx \sqrt{\frac{2F_N q(A-1)B_e}{AP_S^{DC}\exp[-G(t)]}} \tag{25}$$

where F_N is the noise figure of the EDFA, q is the electron charge and B_e is the noise equivalent bandwidth that can be optimized to be equal to the signal bandwidth B_s through digital filtering[6].

IV) Signal-SpBS beating noise (fourth term in Supplementary Eq. (22)). This noise comes from the beating between the SpBS originated from the coded pulse sequence and the probe signal reaching the photodetector. To calculate the noise standard deviation, the electric fields of the probe signal ($\vec{E}_S$) and pump-induced SpBS ($\vec{E}_B$) after the EDFA in front of the detector are firstly described as:

$$\vec{E}_S(t) = \hat{x}\sqrt{A}\,E_s(t)exp(j\omega_o t) \tag{26}$$

$$\vec{E}_B(t) = [\hat{x}\cos\theta + \hat{y}\sin\theta]\sqrt{A}E_b(t)\exp[j(\omega_o + \Delta\omega)t + \Phi_k] \tag{27}$$

where $\hat{x}$ and $\hat{y}$ stand for the local polarization direction of the probe and its respective orthogonal direction; $\Delta\omega$ is the relative optical frequency offset between the probe and SpBS; the angle θ is the relative local polarization rotation of the SpBS with respect to the local probe, which varies in the range of $[0, \pi/2]$; and Φ_k is the random pump-probe phase difference. Thus the detected optical power is:

$$P(t) \propto \left(\vec{E}_B(t) + \vec{E}_S(t)\right)\left(\vec{E}_B^*(t) + \vec{E}_S^*(t)\right) = AE_s^2(t) + AE_b^2(t) + 2AE_s(t)E_b\,(t)\cos(\theta)\cos[\Delta\omega t + \Phi_k] \tag{28}$$

The first right-hand term represents the probe signal reaching the photodetector. The second term is the SpBS component amplified by EDFA, corresponding to a small deterministic signal. The third term however represents the signal-SpBS beating noise, whose normalized standard deviation can be calculated as:

$$\sigma_{s-SpBS} = \frac{\sqrt{\langle\{2AE_s(t)E_b(t)\cos(\theta)\cos[\Delta\omega t + \Phi_k]\}^2\rangle}}{AP_S^{DC}\exp[-G(\mathrm{t})]} = \frac{\sqrt{AE_s^2(t)AE_b^2(t)}}{AP_S^{DC}\exp[-G(\mathrm{t})]} = \sqrt{\frac{P_{SpBS}(t)}{P_S^{DC}\exp[-G(\mathrm{t})]}} \tag{29}$$

where P_{SpBS} represents the optical power of the SpBS light.

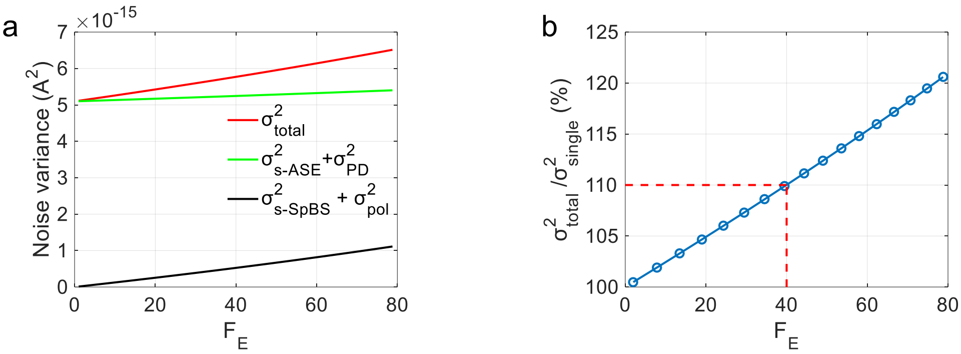

Supplementary Fig. 7. a $\sigma^2_{s-ASE} + \sigma^2_{PD}$, $\sigma^2_{s-SpBS} + \sigma^2_{pol}$ and their summation at the fiber far end as a function of F_E, and **b** Ratio between $\sigma^2_{s-SpBS} + \sigma^2_{pol}$ and σ^2_{single}.

The above-derived expressions enable analytically characterising the noises superposed on the Brillouin gain trace at any fibre location. Here we focus on the fibre far-end, where the SNR is the weakest in single-pulse BOTDA thus the coding gain (SNR improved by coding) is the most demanding. To fully benefit from coding, the total noise at the fibre far-end should remain almost unchanged between single-pulse and coded BOTDA schemes (e.g. tolerating < 10% difference in noise variance), which in turn requires to optimize the energy enhancement factor F_E defined in Results. Supplementary Fig. 7(a) shows the variances of the above-mentioned noises at the fibre far-end as a function of factor F_E, for the case of 2 m spatial resolution BOTDA experiments (described in Results). It can be seen that when $F_E = 1$ (corresponding to the optimised single-pulse Brillouin gain of 2.5%), the signal-ASE beating noise and photo-detection noise ($\sigma^2_{s-ASE} + \sigma^2_{PD}$) are dominant, so that $\sigma^2_{single} = \sigma^2_{s-ASE} + \sigma^2_{PD}$. As F_E gets larger, other noises, i.e. polarization noise and single-SpBS beating noises ($\sigma^2_{pol} + \sigma^2_{s-SpBS}$), gradually increase and become non-negligible. Based on Supplementary Fig. 7(a), the ratio between the noise variances in coded (σ^2_{total}) and single-pulse (σ^2_{single}) BOTDA schemes as a function of F_E, is calculated and shown in Supplementary Fig. 7(b). It can be found that 40 is the optimum (maximum acceptable) value of F_E, which secures a maximum 10% difference between the two noise variances (i.e. noise remains almost unchanged between single-pulse and coded BOTDA schemes).

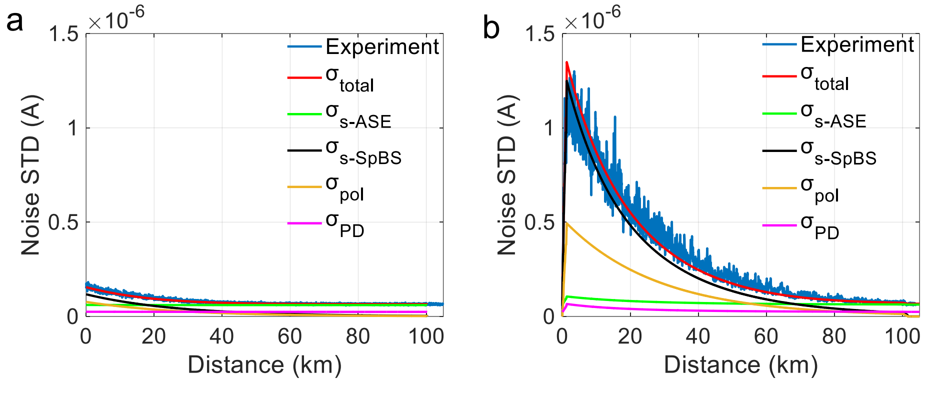

Supplementary Fig. 8. The standard deviations of theoretically predicted noise and measured noise as a function of distance for **a** single-pulse scheme and **b** GO-coded scheme.

In such an ideal case ($F_E \approx 40$), the theoretically calculated standard deviations of different noises and the total noise as a function of the fibre position are all shown in Supplementary Fig. 8(b), in which the theoretical prediction (red curve) of the total noise standard deviations matches well with the experimental result (blue curve), verifying the proposed noise model. Results also demonstrate that all Brillouin-gain dependent noises become negligible close to the fibre far-end (i.e. total noise of coded-BOTDA is approximately equal to that of single-pulse BOTDA shown in Fig. 8(a)), thanks to the proper design of F_E.

To demonstrate that this limitation of F_E (imposed by the additional Brillouin-gain dependent noises) is not

exclusive in the here proposed GO-code, a comparative experiment has been performed with a 255-bit Simplex coded BOTDA. Experimental parameters are the same as those in Results, where F_E is also set to 40. Supplementary Fig. 9(a) shows the decoded Brillouin trace at the resonance peak frequency (averaged 1024 times), compared to single-pulse Brillouin traces averaged by 1024 and 65536 times (representing 9 dB SNR difference between them), exhibiting similar behaviours as the those in Fig. 3(b) obtained by the proposed GO-code. Supplementary Fig. 9(b) illustrates the SNR of each trace versus distance, showing similar level of deterioration as that of GO-code (see Fig. 3(c)). This demonstrates that the negative impact of Brillouin-gain-dependent noises on the SNR enhancement is inherent to coding techniques, regardless of the coding scheme being used.

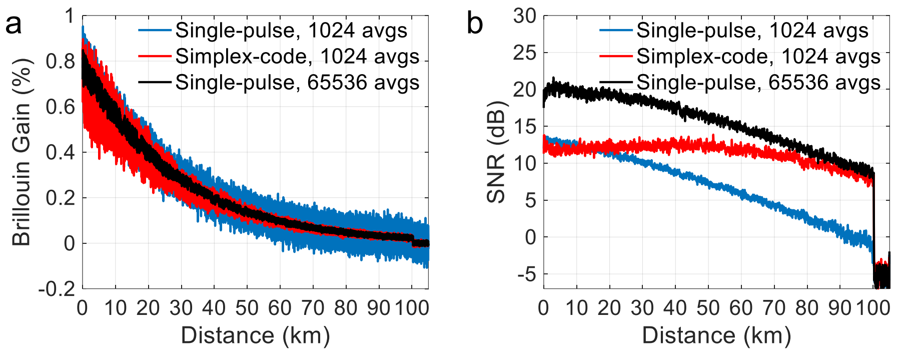

Supplementary Fig. 9. The experimental result of 255-bit Simplex coded BOTDA. **a** The decoded and measured trace along the sensing fibre. **b** SNR profile of each trace.

It is worth to mention that the Brillouin-gain-dependent noises are more detrimental in Cyclic coding. This is because the coded sequences spread all along the fibre, so that the noise induced by the strong signal originating from the fibre near-end will impact on the SNR of the weak signal originating from the fibre far-end. This is experimentally demonstrated with $F_E \approx 40$, as shown in Supplementary Fig. 10(a). By comparing with the noise level in a single-pulse scheme (Supplementary Fig. 8(a)), the noise floor of the Cyclic coding response is globally higher. This means that the theoretical coding gain at the fibre far-end cannot be realised using Cyclic codes (i.e. there is nearly no actual coding gain), as shown in Supplementary Fig. 10(b).

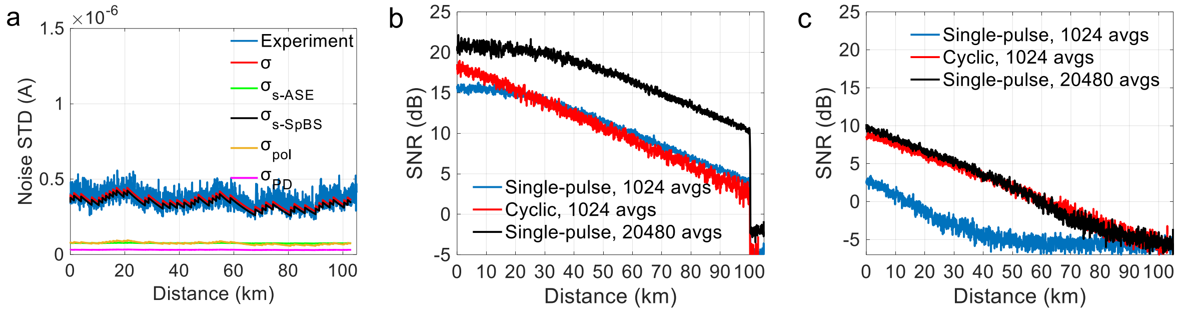

Supplementary Fig. 10. a The standard deviations of theoretically predicted noise and measured noise as a function of distance for Cyclic coding. SNR profile of each trace for Cyclic coding and single pulse with: **b** optimised pulse peak power, **c** only 10 mW pulse peak power

It is also worth to mention that the theoretical coding gain of Cyclic code realised in some literature (e.g. [58] cited in the main text) results from a non-optimised pulse peak power in both single-pulse and Cyclic coding schemes (10 times below the MI threshold). In this case, the signal-dependent noise is much lower, so that the actual coding gain is close the theoretical value; however, this is of limited relevance since the absolute SNR with coding (red curve in Supplementary Fig. 10(c)) is even lower than that of an optimised single-pulse scheme as represented by the blue curve on the previous graph in Supplementary Fig. 10(b).

Note also that, with optimised pulse power, both aperiodic and Cyclic codes cannot bring decisive SNR

improvement in short distance sensing as well. Supplementary Fig. 11(a) and (b) shows the noise profile of aperiodic code and Cyclic code for a 25 km sensing range, respectively, while Supplementary Fig. 11(c) shows the SNR obtained over the entire sensing fibre for both codes as well as for the single pulse case. It can be clearly found that both types of codes cannot provide decisive SNR improvement along the 25 km range, due to the additional signal-dependent noise induced by the code sequences themselves (noise that dominates the measurement). However, it is very relevant to notice that the total noise in the case of Cyclic code remains constant all over the measured trace (as shown in Supplementary Fig. 11(b)), while the total noise reduces with distance for the aperiodic code, as shown in Supplementary Fig. 11(a). This behaviour is essentially due to the pulse distribution over the sensing fibre, explaining the higher SNR improvement provided by aperiodic codes at longer sensing ranges compared to Cyclic coding as demonstrated in Supplementary Figs. 9 and 10.

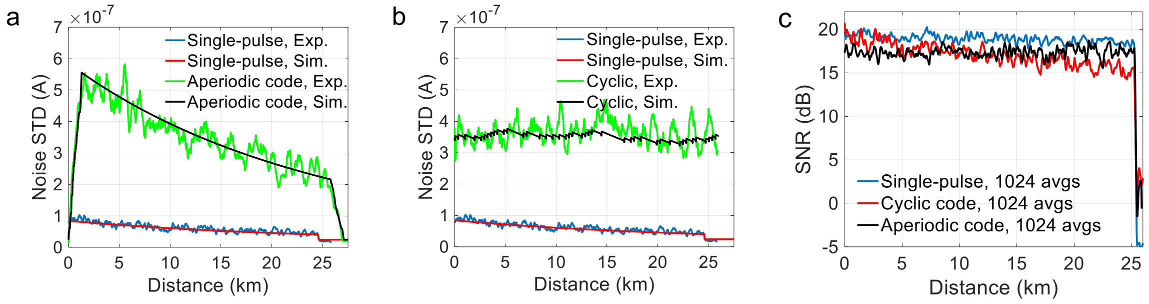

Supplementary Fig. 11. Noise profiles for **a** aperiodic code and **b** cyclic code. **c** SNR profile over distance for single-pulse, aperiodic code and cyclic code cases.

All results indicate that there is no benefit brought by Cyclic coding when comparing with a fully optimised single-pulse scheme for any sensing range. Note however that, for a non-optimised pulse power, cyclic coding can be effective as demonstrated by previous publications, though this non-optimised condition has not yet proved to bring any global advantage in real conditions.

Supplementary note 4. Noise analysis of coded ROTDR system

In coded ROTDR systems, the use of coding increases the spontaneous Raman backscattering power, which may lead to a non-negligible shot-noise level in photo-detector, compromising the expected coding gain. This note describes the limitations imposed by these two phenomena and presents a model that can quantitatively define the optimal energy enhancement factor F_E.

Considering the limitations imposed by amplified spontaneous Raman scattering (ASpRS), it must be noted that each code pulse simultaneously generates forward and backward Raman scattering components. In the case of a single pulse, the forward Stokes and anti-Stokes components interact with the pump pulse over an effective optical fibre length determined by the walk-off distance and optical losses. In the case of coding, return-to-zero format is required to avoid interactions between code pulses and Raman components generated by adjacent pulses in the sequence, which may occur due to group velocity differences among spectral components. Under this condition, the threshold power for forward ASpRS turns out to be equivalent to the single pulse case, i.e. being around 1 W for metre-scale spatial resolutions.

Having fixed the peak pump power (e.g. to 1 W as in this case), the optimal energy enhancement factor F_E is primarily defined by the photo-detection noise. Indeed, a good balance between thermal noise and shot noise must be achieved to obtained the real benefits from coding, i.e. to enhance the SNR by the expected coding gain. While the thermal noise of APD is constant at a given temperature, shot noise increases with the input optical power. The variance of the shot noise σ_s^2 in an APD detecting the coded anti-Stokes SpRS signal, and ignoring dark current, is given by[7]:

$$\sigma_s^2(z) = 2qM^2F_ARF_EP_{single}(z)B \tag{30}$$

where q is the elementary charge, M is the mean APD gain factor, F_A is the APD excess noise factor, R is the APD responsivity, $P_{single}(z)$ is the single-pulse power response of the anti-Stokes SpRS, and B is the electrical bandwidth. Supplementary Eq. (30) is experimentally validated for the case of 2 m SR, as shown in Supplementary Fig. 12(a), where the variance of the equivalent APD noise in the single-pulse case ($F_E = 1$) is characterised as $1.6 * 10^{-3}\ V^2$, corresponding to the contribution of thermal noise. Note that this behaviour is enhanced in the case of APD when compared to PIN photodetectors, due to the impact of the avalanche amplification process and induced excess noise (i.e. because $M^2F_A \gg 1$).

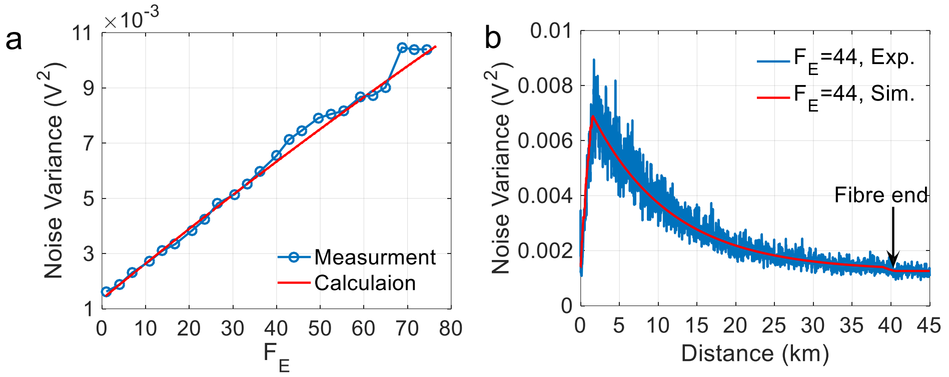

Supplementary Fig. 12. a Measured and calculated noise variance as a function of the energy enhancement factor. **b** Measured and simulated noise of coded ROTDR trace as a function of distance.

Since the total anti-Stokes SpRS power reaching the detector reduces as a function of distance along the sensing fibre, the additional noise introduced over the ROTDR traces decreases with distance. To fully benefit from coding, the total noise at the fibre far-end should remain almost unchanged between single-pulse and coded ROTDR schemes (i.e., noise near the fibre-end in both schemes should be dominated by thermal noise), requiring to optimise the energy enhancement factor F_E. Based on Supplementary Eq. (30) and considering the fibre attenuation, $F_E = 44$ is calculated for the case of 2 m SR and the 39 km long sensing fibre used in Results, as shown in Supplementary Fig. 12(b). It can be determined that the noise near the far fibre-end (between 35 and 39 km) is highly dominated by the thermal noise, since the noise variance over this section is visually identical to that outside the fibre.

Supplementary note 5. Experimental results with 1 m spatial resolution

Similar to the demonstration of GO-coded BOTDA with 2 m spatial resolution (SR), as shown by Fig. 3 in Results, experiments are here carried out at 1 m SR. This enables the use of a longer code sequence, thus expecting a larger SNR improvement. Due to the broadened Brillouin spectral response when using 1 m SR, a wider frequency scan (300 scans) with a frequency step of 1 MHz is performed. Temporal Brillouin traces are averaged 1024 times, leading to a total measurement time of ~5.7 min. The optimised single-pulse Brillouin gain peak is 0.5%, determining an optimal energy enhancement factor $F_E = 200$. The decaying amplitude imposed by the EDFA gain saturation leads to an optimal 723-bit GO-code sequence that offers a theoretically evaluated coding gain of 9.3 dB, being only 0.7 dB lower than the standard reference coding gain (10 dB, $\sqrt{(200/2)} = 10$ times in linear scale). Similar to the demonstration performed at 2 m SR, relevant results are shown in Supplementary Fig. 13(a)-(c), respectively, where the reference curves (black) are obtained using the single-pulse scheme with 74182 averages, representing a reference SNR improvement equal to the expected coding gain ($10lg\sqrt{74182/1024} = 9.3\ dB$). As anticipated, the additional Brillouin-induced noises still compromise the performance of GO-code measurements at the fibre near-end, while showing a negligible effect at the fibre far-end.

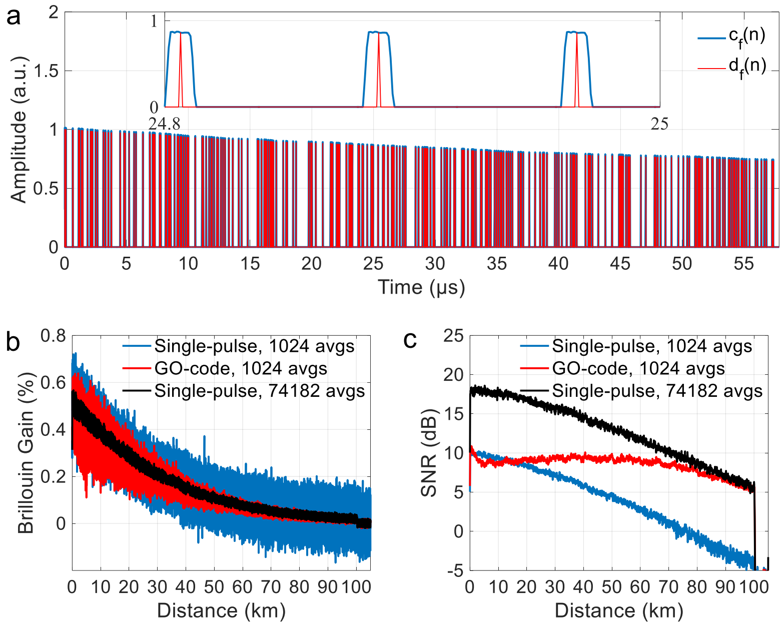

Supplementary Fig. 13. Experimental results of BOTDA with 1 m SR. **a** 723-bit normalised coding sequence $c_f(n)$ measured at the fibre input (blue) and the retrieved $d_f(n)$ (red). Inset: Zoom-in of normalised $c_f(n)$ and $d_f(n)$ over a time span from 24.8 μs to 25 μs. **b** Temporal BOTDA gain traces at fibre Brillouin resonance. **c** SNR profiles over the entire sensing fibre.

Measurements of ROTDR with 1 m SR are then performed. In this case, the optimised energy enhancement factor F_E is determined to be 150, leading to a 450-bit code that takes into account the decaying trend of the sequence envelop. The GO-code found by DGA exhibits a theoretical coding gain of G_c = 8.67 dB, being 0.71 dB below the standard reference coding gain G_r = 9.38 dB. Similar to the demonstrations in Fig. 4, relevant results associated to 1 m SR are illustrated in Supplementary Fig. 14(a)-(c). The SNR improvement at the fibre far-end is found to be 8.63 dB, perfectly matching the theoretical value.

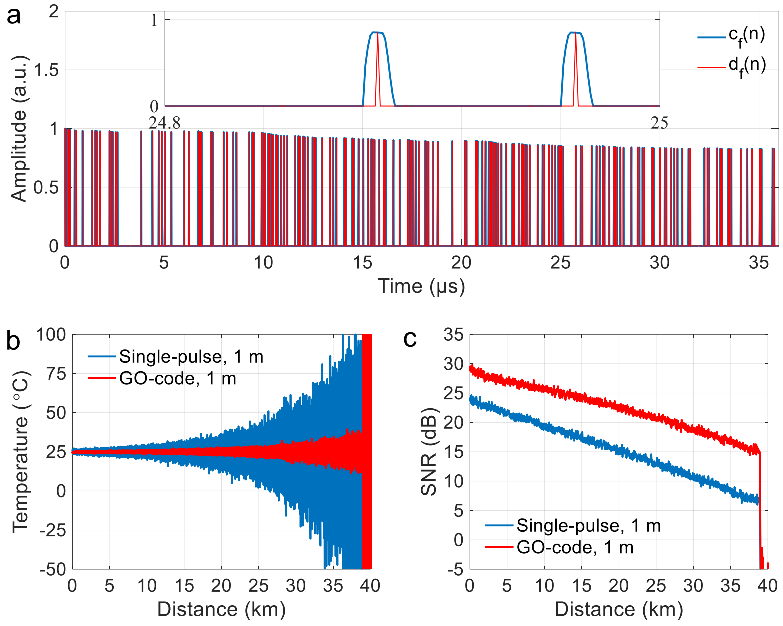

Supplementary Fig. 14. Experimental results of ROTDR with 1 m SR. **a** 450-bit normalised coding sequence $c_f(n)$ measured at the fibre input (blue) and the retrieved $d_f(n)$ (red). Inset: Zoom-in of normalised $c_f(n)$ and $d_f(n)$ over a time span from 24.8 μs to 25 μs. **b** Retrieved temperature profiles for single pulse (blue) and GO-code (red). **c** SNR profiles over the entire sensing fibre.

Supplementary References

1. Proakis, J. G. & Manolakis, D. G. *Digital Signal Processing: Principles, Algorithms, and Applications* 4th edn (Pearson Education, Inc., 2007).
2. Golay, M. Sieves for low autocorrelation binary sequences. *IEEE Transactions on Information Theory* **23**, 43–51 (1977).
3. Yang, Z., Li, Z., Zaslawski, S., Thévenaz, L. & Soto, M. A. Design rules for optimizing unipolar coded Brillouin optical time-domain analyzers. *Opt. Express* **26,** 16505–16523 (2018).
4. Soto, M. A, Tur, M., Lopez-Gil, A., González-Herráez, M. & Thévenaz, L. Polarisation pulling in Brillouin optical time-domain analysers. *25th Optical Fiber Sensors Conference (OFS)*,1-4 (2017). DOI: 10.1117/12.2267649.
5. Wang, S., Yang, Z, & Soto, M. A. & Thévenaz, L. Study on the signal-to-noise ratio of Brillouin optical-time domain analyzers. *Opt. Express* **28,** 19864–19876 (2020).
6. Zaslawski, S., Yang Z., & Thévenaz, L. On the 2D Post-Processing of Brillouin Optical Time-Domain Analysis. *J. Lightwave Technol.* https://doi.org/10.1109/JLT.2020.2967091 (2020).
7. Saleh, B. E. A. & Teich, M. C. Fundamentals of Photonics Ch. 18, 784–803 (Wiley, 2007).
