# Supplementary material for: Genetic-optimised aperiodic code for distributed optical fibre sensors
Source: Nat Commun. 2020 Nov 13;11:5774. doi: 10.1038/s41467-020-19201-1 (PMC7666181; doi:10.1038/s41467-020-19201-1)
Supplement: Supplementary file 3 — Description of Additional Supplementary Files [file 41467_2020_19201_MOESM3_ESM.pdf]

Description of Additional Supplementary Files

File name: Supplementary Movie 1
Description: “The video shows realtime measurement of fast heating and cooling processes, using the proposed GO-coded Raman optical time-domain reflectometry, over two fibre sections (2 m and 5 m, separated by a 5 m unperturbed section) at the far-end of a 10.2 km sensing fibre. The sampling time is 1 second, and the inset figures are obtained by on-line real-time decoding (synchronised with the measurements). The video is displayed with 2X speed.”
